# NMR-Based Detection of Hydrogen/Deuterium Exchange in Liposome-Embedded Membrane Proteins

**DOI:** 10.1371/journal.pone.0112374

**Published:** 2014-11-06

**Authors:** Xuejun Yao, Ulrich H. N. Dürr, Zrinka Gattin, Yvonne Laukat, Rhagavendran L. Narayanan, Ann-Kathrin Brückner, Chris Meisinger, Adam Lange, Stefan Becker, Markus Zweckstetter

**Affiliations:** 1 Max Planck Institute for Biophysical Chemistry, Göttingen, Germany; 2 Institut für Biochemie und Molekularbiologie, ZBMZ and BIOSS Centre for Biological Signalling Studies, Universität Freiburg, Freiburg, Germany; 3 German Center for Neurodegenerative Diseases (DZNE), Goöttingen, Germany; 4 Center for Nanoscale Microscopy and Molecular Physiology of the Brain (CNMPB), University Medical Center, Göttingen, Germany; George Washington University, United States of America

## Abstract

Membrane proteins play key roles in biology. Determination of their structure in a membrane environment, however, is highly challenging. To address this challenge, we developed an approach that couples hydrogen/deuterium exchange of membrane proteins to rapid unfolding and detection by solution-state NMR spectroscopy. We show that the method allows analysis of the solvent protection of single residues in liposome-embedded proteins such as the 349-residue Tom40, the major protein translocation pore in the outer mitochondrial membrane, which has resisted structural analysis for many years.

## Introduction

Membrane proteins have important biological functions and are the targets of over 50% of all modern medicinal drugs. However, only a small number of membrane protein structures have been solved up to now (http://blanco.biomol.uci.edu/mpstruc/#Latest). Structure determination of membrane proteins by either nuclear magnetic resonance (NMR) spectroscopy or X-ray crystallography is complicated by the need to solubilize membrane proteins in native-like environments [1]. In addition, solution-state NMR spectroscopy of membrane proteins relies on the ability to find detergents, bicelles or nanodiscs, in which the native structure of the protein is retained and relaxation losses are minimized [2]. Solid-state NMR spectroscopy on the other hand can investigate membrane proteins reconstituted into liposomes or uniformly oriented bilayers [3]–[9], but requires sufficient spectral quality to enable sequence-specific resonance assignment and structure determination.

Hydrogen/Deuterium (H/D) exchange has long been used to probe protein structures [10]. The success of H/D exchange is based on the strong influence of hydrogen bonds on amide proton exchange rates. Due to its power to provide single-residue information, NMR spectroscopy is optimally suited to monitor site-specific H/D exchange rates of proteins [10]–[13]. For membrane proteins, H/D exchange rates are particularly important, as it is often difficult to obtain a sufficient number of distance restraints [14]–[16]. In addition, H/D exchange coupled to solution-state NMR is useful for high-molecular weight systems that would otherwise not be accessible to solution-state NMR. A particularly important application is the investigation of the structure of protein aggregates, in which the H/D exchange profile of the protein aggregate is detected with the help of the denatured monomer [17]–[19]. H/D exchange in membrane proteins can be also analyzed by mass spectrometry [20], [21], although generally not at the residue resolution achievable by NMR spectroscopy. In addition, a solid-state NMR H/D exchange experiment performed on a helical membrane protein showed that the amide protons in an amphipathic helix are more slowly exchanging than those in the transmembrane helix in a four helix bundle with an aqueous pore [22].

Here we demonstrate that the solvent protection of single residues in liposome-embedded transmembrane proteins can be studied using solution-state NMR spectroscopy. Using a dedicated H/D exchange protocol, the information of the membrane-embedded state is transferred to the denatured state and analyzed using multidimensional NMR. The method is applied to the 349-residue protein Tom40 that forms the protein translocation pore in the outer mitochondrial membrane and has resisted structural analysis for many years [23]–[25].

## Materials and Methods

### Sample preparation

Tom40 from *neurospora crassa* (ncTom40) was expressed, refolded and purified as previously described [25]. ^15^N- and ^13^C-labelled protein was expressed in minimal medium with ^15^NH_4_Cl as nitrogen source and ^13^C_6_-D-glucose as carbon source. Amino acid-selective labelled ncTom40 protein was expressed according to a recently published protocol [26]. For solid-state NMR measurements and H/D exchange the protein was reconstituted in DMPC at a protein/lipid ratio of 1∶50 (mol/mol). H/D exchange for ncTom40 in liposomes was performed in 10 mM MOPS, 10 mM KCl, pD 7.0, 100% D_2_O. Back-exchange was monitored in a dissolution buffer containing 4 M guanidinium thiocyanate (GdnSCN), 0.4% formic acid, pD 2.5.

### NMR spectroscopy

Experiments were carried on Bruker 800 and 900 MHz spectrometer equipped with cryogenic probes. 6D HNCOCANH and 5D CBCACONH automated projection spectroscopy (APSY) experiments were recorded at 295K and 278K, while 7D HNCO(CA)CBCANH and 5D CBCACONH were measured at 310K [27]–[29]. APSY spectra were processed using PROSA [30]. Peaks on each projection spectrum were picked and the final peak list was calculated using GAPRO [28]. A 3D HNN experiment [31] was recorded at 278K. Assignment was performed in an iterative manner using MARS [32], manual inspection of the 3D HNN experiment [31] and HSQCs of amino acid selectively ^15^N-labelled samples (Table S1). Back-exchange in dissolution buffer was monitored using two-dimensional [^1^H, ^15^N]-HSQC spectra recorded with a Bruker BEST-HSQC [33] pulse sequence at 278K with a recycle delay of 0.5s. HSQCs were processed and analyzed using NMRPipe [34] and Sparky 3 (University of California, San Francisco).

Solid-state NMR experiments were conducted using 4 mm triple-resonance magic-angle spinning (MAS) probeheads at a static magnetic field of 18.8 T and using 8.33 kHz MAS. Sample temperatures were +5°C for ^13^C-^13^C proton-driven spin diffusion (PDSD) correlation experiments and +15°C for INEPT-type experiments. Initial cross-polarization time for PDSD was set to 600 µs for ^1^H-^13^C transfer. ^13^C-^13^C mixing was accomplished by PDSD for 15 ms to obtain intra-residual correlations. The ^13^C-^13^C INEPT-TOBSY [35] correlation spectrum was recorded with decoupling field strength of 2.5 kHz.

## Results

**Figure 1:**
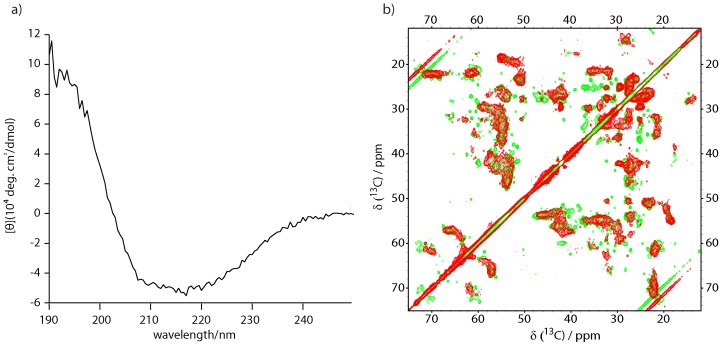
Recombinant ncTom40 has a β-barrel structure. (a) Far UV CD spectra of ncTom40 in decylmaltoside. (b) Superposition of ^13^C-^13^C proton driven spin diffusion spectra of ncTom40 (red) and hVDAC1 (green; reproduced from [4]), both in DMPC liposomes. The mixing time was 15 ms.

^13^C/^15^N-labeled ncTom40 was prepared recombinantly and subjected to refolding in detergent. Circular dichroism spectra of our ncTom40 preparation in decylmaltoside (Figure 1a) closely resembled previous CD spectra of recombinantly produced and refolded ncTom40, which had been demonstrated to be functional [25]. Using different algorithms the β-structure content had been estimated to be approximately 30–40%, consistent with a β-barrel structure (the expected percentage of β-structure in the 349-residue ncTom40 is 44% assuming the presence of 19 strands with a length of 8 residues on average) [25]. Despite screening many different conditions, however, it was not possible to obtain high-quality solution NMR spectra of ncTom40 solubilized in detergent (Figure S1). This might be due to the strong propensity of Tom40 to form homooligomers [23].

Next, ncTom40 was reconstituted into 1,2-Dimyristoyl-***sn***-Glycero-3-Phosphocholine (DMPC) liposomes. In a two-dimensional [^13^C,^13^C] solid-state NMR spectrum of liposome-embedded ncTom40 a large number of cross-peaks was observed (Figure 1b). The NMR signal distribution was similar to that previously observed for the 283-residue human voltage-dependent anion channel (isoform 1; hVDAC1), which shares a 30% sequence similarity with ncTom40. The structure of hVDAC1 is composed of 19 β-strands and an N-terminal α-helix [36]–[38]. The solid-state NMR spectrum of ncTom40 is of lower quality than that of hVDAC1 (Figure 1b), but contained a larger number of defined cross peaks than isoform 2 of VDAC (hVDAC2), both of which were shown to be functional [4], [39], [40]. The similarity of the ^13^C-^13^C correlation spectra of ncTom40, hVDAC1 and hVDAC2 (Figure 1b and [39]) together with the CD profile of refolded ncTom40 (Figure 1a and [25]) indicates that ncTom40 folds into a β-barrel in both detergent and liposomes. Notably, neither for hVDAC1/2 or a β-barrel protein of similar size, the sequence- specific resonance assignment of its β-barrel when inserted into liposomes has been reported till date. This highlights the need to develop methods that allow characterization of the structure of large liposome-embedded proteins at single-residue resolution.

**Figure 2:**
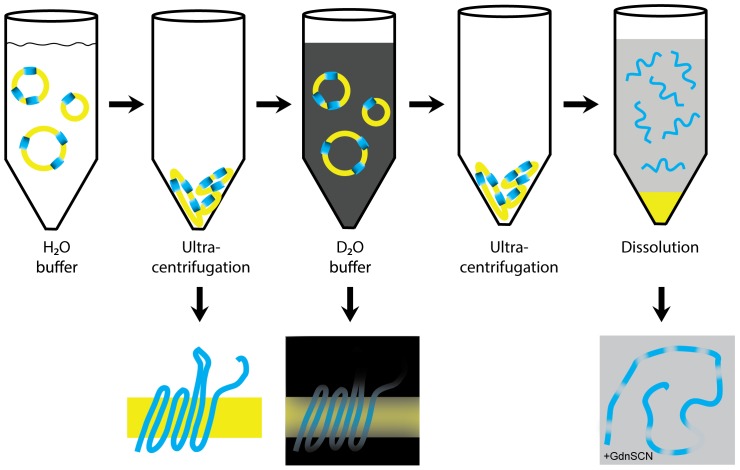
Scheme illustrating the H/D exchange strategy developed for membrane proteins (blue) reconstituted into liposomes (yellow). A white color indicates H_2_O buffer, black color 100% D_2_O buffer and grey color the dissolution buffer, which contains 4 M GdnSCN. During the incubation period in 100% D_2_O solvent exposed residues will exchange amide protons against deuterium (lower row, middle panel). They will therefore not be visible in the denatured monomer (lower right panel).

To address this need, we designed the H/D exchange approach outlined in Figure 2. The protein containing liposomes are centrifuged at 168,000×g for 1 hr at 4°C. The pellet is then transferred to 100% D_2_O and incubated with agitation for 10 minutes at room temperature. During this time, amide protons will undergo exchange. At the end of the incubation time, the pellet is collected through centrifugation for 15 minutes and put into dissolution buffer that contains 4 M GdnSCN, 0.4% formic acid at pD 2.5 [41], with either 75% or 100% D_2_O. In the dissolution buffer lipids immediately precipitate, while the protein stays in solution. Subsequently, [^1^H, ^15^N] heteronuclear single quantum coherence (HSQC) spectra are recorded (Figure S2) [41]. The dead time before starting the NMR experiment was approximately 20 minutes. To minimize the influence of back-exchange [42], the temperature was set to 278K and the experiments were recorded using the BEST scheme [12], [33].

**Figure 3:**
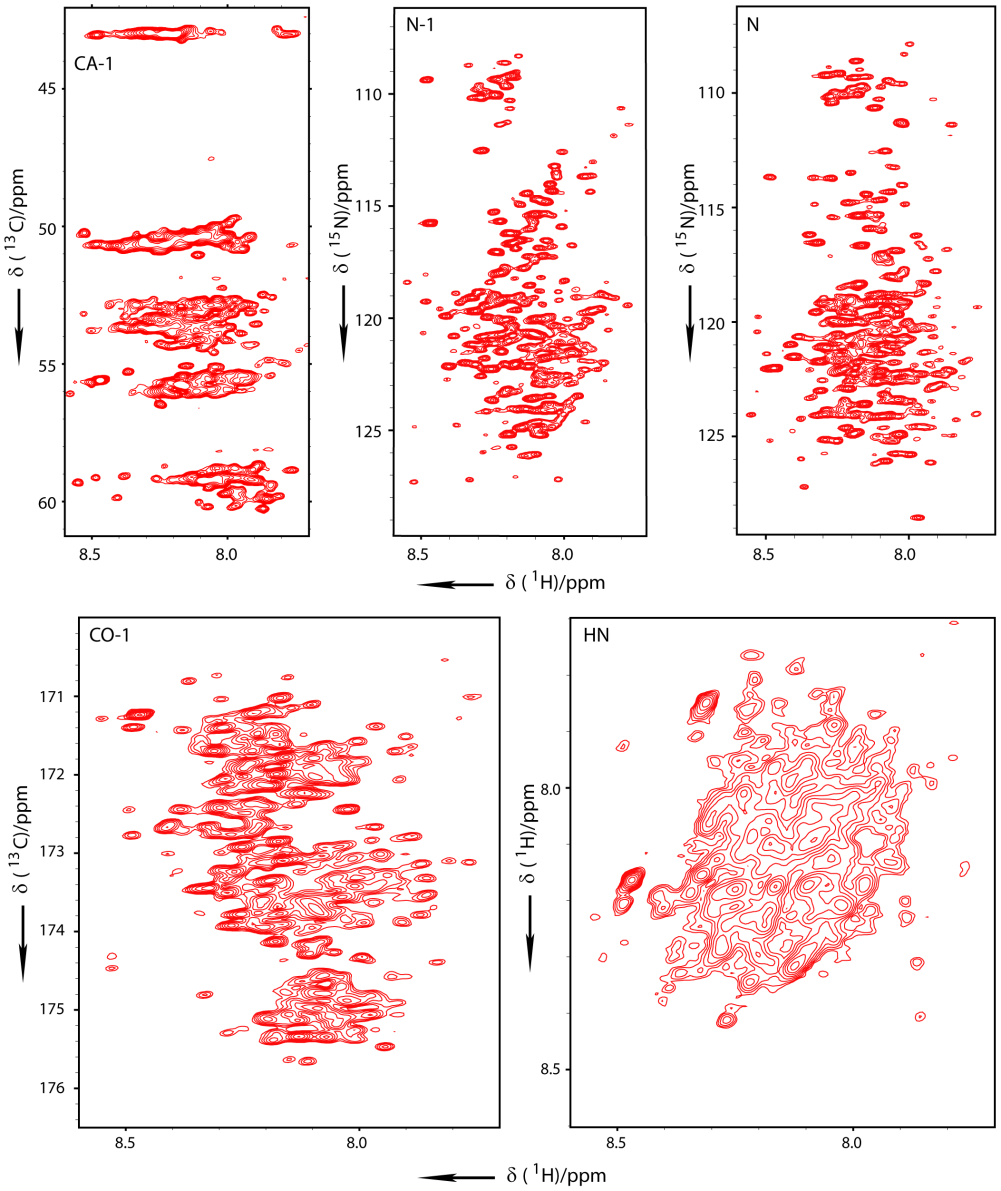
High-dimensional NMR experiments for assignment of ncTom40 in the denatured state. 2D projections (CA_i-1_-HN_i-1_, N_i-1_- HN_i-1_, N_i_-HN_i-1_, CO_i-1_-HN_i-1_, HN_i_-HN_i-1_) of the 6D APSY-HNCOCANH experiment, recorded on ncTom40 in 4 M GdnSCN, 0.4% formic acid. The measurement temperature was 278 K.

To obtain the sequence-specific resonance assignment of the 349 residues of ncTom40 in the dissolution buffer, we performed APSY experiments and assignment using the program MARS [28], [32], [43]. APSY enables the measurement of high-dimensional spectra, reducing NMR signal overlap [27], [28]. High-dimensional APSY experiments [27], [28] were recorded at 278 K, 295 K and 310 K on ^13^C/^15^N-labeled ncTom40 in 4 M GdnSCN, 0.4% formic acid (Figure 3). Most residue-specific assignments were obtained at 310 K (see Table 1), consistent with the increase in relaxation times at higher temperature. To further increase the assignment coverage, six-dimensional peak lists from high and low temperature spectra were automatically matched [43]. In combination with the sequential connectivity found in the APSY spectra recorded at 278 K and manual inspection of a 3D HNN experiment [31] 326 out of 339 non-proline residues of ncTom40 could be assigned. ncTom40 in GdnSCN is thus one of the largest unfolded proteins for which the backbone assignment was obtained [43]–[46].

**Table 1:** APSY experiments recorded at different temperatures and assignments obtained for denatured ncTom40 (339 non-proline residues) by MARS [31].

Temperature	Experiments	Numbers of assigned residues	
		Reliable	Low reliable
310 K	7D HNCO(CA)CBCANH	312	11
	5D CBCACONH		
295 K	6D HNCOCANH	249	26
	5D CBCACONH		
278 K	6D HNCOCANH	128	58
	5D CBCACONH		

Assignments classified by MARS as low are not reliable and were excluded from further analysis.

**Figure 4:**
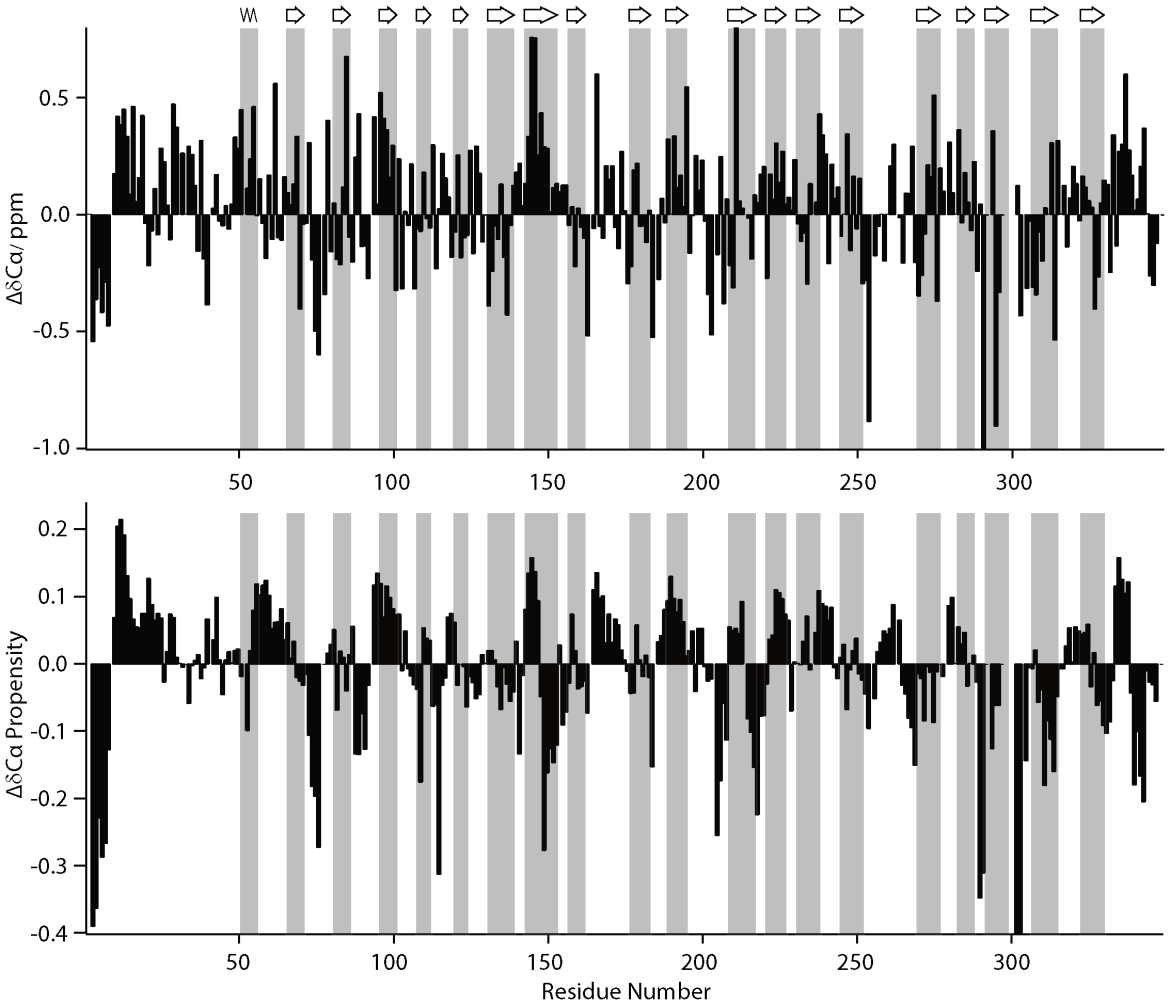
Cα secondary chemical shifts (upper chart) and Cα secondary structure propensities (lower chart) of ncTom40 obtained from APSY experiments recorded at 295K. Secondary structure propensities were calculated using SSP [42]. The predicted topology of ncTom40 is shown on top with secondary structure elements highlighted in grey. Only assignments classified by MARS [27] as reliable were used.

The residue-specific assignment of ncTom40 in 4 M GdnSCN allows the analysis of residual structure in the denatured state. This is important as the presence of residual structure in the dissolution buffer might influence the back-exchange process. Comparison of carbon chemical shifts in ncTom40 in 4 M GdnSCN with random coil values showed that for most residues the secondary structure propensity is below 0.2 [47]. No region exists where more than three consecutive residues exceed the secondary structure propensity value of 0.3 (Figure 4), indicating that very little residual structure, which could potentially influence back-exchange, is present in ncTom40 in 4 M GdnSCN.

**Figure 5:**
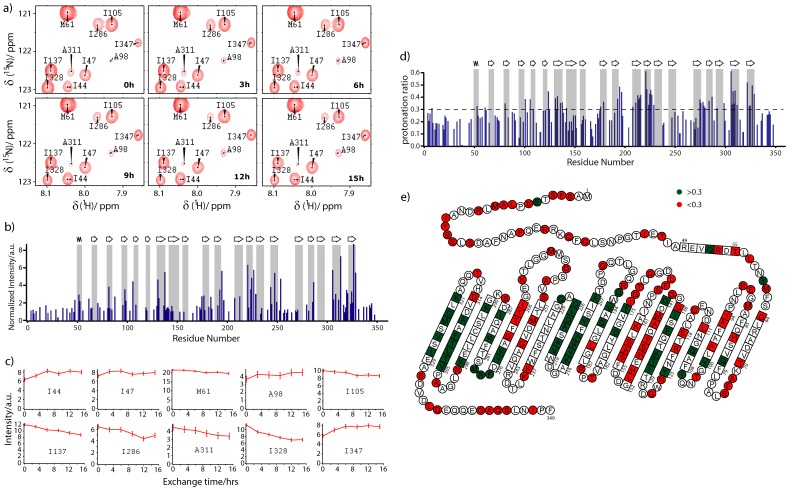
Structural characterization of liposome-embedded ncTom40 by H/D exchange coupled to solution-state NMR. (a) Enlarged spectral regions of [^1^H, ^15^N]-HSQC spectra at increasing back-exchange times. To reduce signal overlap, ncTom40 was selectively ^15^N-labeled at ALA, HIS, ILE, MET, THR. Time points indicate the time after start of the first HSQC. The dissolution buffer contained 75% D_2_O. Sequence-specific resonance assignments are indicated. (b) Sequence-specific signal intensities in the first HSQC after dissolution in 100% D_2_O buffer. (c) NMR signal intensity change of residues in panel (a) during back-exchange in 75% D_2_O. Intensity values were normalized on the basis of the noise level in the spectra. Error bars are based on signal-to-noise. (d) Protonation ratios for residues of Tom40 at the beginning of back exchange. (e) Protonation ratios shown in (d) were mapped onto the topology model of ncTom40, which was predicted on the basis of its homology to hVDAC1. Residues predicted to be in a β-strand or α-helix are boxed. Green-shaded (red-shaded) residues have protonation ratios larger (lower) than 0.3. Residues shown in white were not analyzed due to signal overlap, low signal-to-noise or missing resonance assignment.

Although 326 residues could be assigned in high-dimensional spectra, signals were severely overlapped in two-dimensional [^1^H, ^15^N]-HSQC spectra (Figure S2). To allow analysis of the back-exchange curves of a large number of residues, amino acid specific labeling was used (Table S1). The combination of amino acid types in each sample was chosen to minimize NMR signal overlap (Figure S3). Figure 5a shows the isoleucine region of the [^1^H, ^15^N]-HSQC spectra recorded on a selectively A/H/I/M/T ^15^N-labeled sample of ncTom40 that had been exposed to the HD exchange protocol using 75% D_2_O dissolution buffer. Six HSQC spectra were measured consecutively, with each spectrum recorded for three hours. In the first spectrum (labelled “0h” in Figure 5a) the signal intensity varied between different residues. For example, the signal intensity of I347 is lower than that of I137 and I328, suggesting that the amide proton of I347 has undergone more exchange during the H/D exchange period and is therefore less protected from solvent in the native state than I137 and I328. A distinct solvent protection along the sequence of ncTom40 was also supported by normalized signal intensities in the first HSQC spectrum after dissolution in a 100% D_2_O dissolution buffer (Figure 5b).

Identification of slow and fast solvent exchange in the native state on the basis of signal intensities in the first spectrum after dissolution relies on the assumption that (i) the dead time before starting the NMR experiment is zero and (ii) that the intrinsic exchange rates of different residues are identical. To overcome these limitations we further analysed NMR signal intensities during back-exchange in 75% D_2_O dissolution buffer (Figure 5c). When amide protons slowly exchanged with deuterium in the liposome state and therefore retained a protonation level of more than 25%, the cross peak intensity decays as for example for residues I105, I137, I286, A311 and I328 (Figure 5c). In contrast, amide protons, which rapidly exchanged during the forward exchange period, will have low protonation levels when the dissolution is started. When their protonation levels are below 25%, they therefore will gain signal intensity due to back-exchange in the dissolution buffer. This is for example seen for I44, I47, A98 and I347 (Figure 5c). Notably, when the back-exchange rate in the dissolution buffer is very fast the signal intensity will remain constant in consecutive HSQCs. These residues will therefore be excluded from the analysis. Analysis of the back-exchange curves has the further advantage, that the protonation level at time 0 in the dissolving buffer can be estimated by an exponential decay curve that takes into account the dead time for the setup of the NMR experiment (approximately 20 minutes) [41]. In this way, estimates for the protonation level at the end of the solvent exchange time in the native state become accessible. The approach is distinct from H/D exchange measurements, in which increasing durations of forward exchange are used to determine protection factors with the following advantages: (i) at the end of the back-exchange the protonation level is 75% providing an internal reference that can be used to back-calculate the protonation level at the end of the forward exchange time; (ii) different samples can be compared on the basis of the internal reference; (iii) variations due to sample differences and dead time in NMR experiments are minimized.

Because a larger number of amino acid specific labeled samples was used for dissolution in 75% D_2_O buffer, more residues could be analysed when compared to dissolution in 100% D_2_O (Table S1 and Figure 5b, d). At the same time, some residues, which are present in Figure 5b, were excluded from the back-exchange analysis as their back-exchange curves could not be described by a single exponential function. Based on the 145 residues with non-overlapping cross-peaks, we found that within the first ∼50 residues the estimated protonation levels were close to or below 0.3 (Figure 5d). A high solvent exposure of the N- and C-terminal regions of liposome-embedded ncTom40 is in agreement with the signal intensities in the first HSQC spectrum after dissolution in 100% D_2_O (Figure 5b) and with sequence-based analysis that predicts the first 50 and last 15 residues of ncTom40 to be disordered (Figure 5e). In between residues 51–330, exponential fits to the back-exchange curves resulted in protonation values exceeding 0.3 for residues 52, 60, 80, 100, 105, 108, 123, 130–132, 135, 137, 141,175, 178, 192, 194, 196, 207, 210, 212–215, 220–221,223–226, 228, 274–275, 277–279, 283, 286, 291, 298, 305–309, 311, 321, 323–324, 326, 328 (Figure 5d, e).

Both the signal intensities in the first HSQC spectrum after dissolution in 100% D_2_O buffer (Figure 5b) and the protonation levels estimated from back-exchange curves in 75% D_2_O buffer (Figure 5d, e) identified residues that exchange more slowly with solvent when ncTom40 is embedded into liposomes. As the slow solvent exchange is not restricted to a specific amino acid type, it suggests that the observed variable degrees of protection are not purely caused by differences in intrinsic solvent exchange rates. In addition, certain amino acid types such as isoleucine are found protected when located in the central part of ncTom40, but solvent accessible when located near the N- or C-terminus. H/D exchange rates in liposome-embedded ncTom40 might be influenced by several other factors such as the type of secondary structure making a quantitative interpretation of the detected protonation levels challenging. However, a multitude of studies have shown that the strongest effect on solvent exchange is exerted by the presence and absence of hydrogen bonds. The higher protonation values – that is less efficient H/D exchange– observed for distinct residues in liposome-embedded ncTom40 therefore suggest that these residues are hydrogen-bonded. Notably, the fact that Tom40 forms a water-filled channel [48], the membrane insertion itself is unlikely to be responsible for the observed decrease in solvent exchange of membrane-embedded ncTom40. Moreover, a few residues, which are not predicted to be part of a β-strand or the N-terminal α-helix such as Y60 and ^277^FRM^279^, appear partially protected from H/D exchange (Fig. 5e), suggesting that hydrogen bonds stabilize the conformation in these region of the protein.

CD spectroscopy and solid-state NMR spectroscopy supports a β-barrel structure of ncTom40 (Figure 1). In addition, secondary structure analysis of ncTom40 on the basis of the known 3D structure of hVDAC1[36]–[38] using the software iTasser[49] predicts 19 β-strands and an N-terminal α-helix in ncTom40 (Figure 5e). The residues that were identified to be solvent protected support the location of several of the predicted β-strands (Figure 5d, e). These are particularly the predicted strands β6 (residues 129–138), β12 (residues 219–226), β18 (residues 305–314) and β19 (residues 321–329), which contains the membrane insertion signal of Tom40 [50]. For the other predicted strands as well as for the predicted α-helix at the N-terminus, the H/D exchange measurements do not provide clear support. This is partially due to the fact that only a subset of residues could be analyzed due to signal overlap and missing resonance assignments. At the same time, however, it is surprising that the amide protons of some of the predicted β-strands rapidly exchanged with solvent. For example, no amide proton with slow H/D exchange was observed for residues 142–161, the residues that are predicted to comprise β7 and β8. This could have several reasons such as the involvement in oligomer formation (in ^145^QFEHEH^150^ every other residue faces the lipid environment such that at best two hydrophilic histidine residues point into the lipid environment) or the presence of chemical exchange [51], [52]. For example, we previously showed that the N-terminal part of the β-barrel of hVDAC1 experiences dynamics on multiple time scales [53]. The amide protons in this region of the hVDAC1 barrel rapidly exchange with solvent, despite the fact that NOE contacts proof the presence of β-strands and defined β-strands were observed in the crystal structure of mouse VDAC1 [38]. H/D exchange measurements performed on detergent solubilized OmpA also had shown that its barrel does not behave like a solid block, but some strands are more mobile or accessible than others [54]. In addition, conformational exchange in the liposome-embedded state could cause a multi-exponential solvent exchange behavior. Finally, the number and/or exact location of the secondary structure elements predicted by iTasser may not be fully correct and therefore account for the differences between the H/D profile and the predicted location of β-strands.

## Conclusions

We demonstrated that the solvent accessibility of single residues in large transmembrane proteins that are embedded in the near native environment of liposomes can be probed using a combination of H/D exchange and solution-state NMR spectroscopy. The method is applicable to highly challenging systems such as Tom40, which has resisted structural analysis by X-ray crystallography and NMR spectroscopy for many years.

## Supporting Information

Figure S1 **Two-dimensional [^1^H,^15^N]-HSQC spectrum of uniformly ^15^N-labeled ncTom40 reconstituted into lauryldimethylamineoxide.**
(TIF)

Figure S2 **Two-dimensional [^1^H,^15^N]-HSQC spectrum of uniformly ^15^N-labeled ncTom40 in dissolution buffer containing 4M GdnSCN, 0.4% formic acid.**
(TIF)

Figure S3 **[^1^H,^15^N]-HSQC spectra of ncTom40 with amino acid selective ^15^N-labeling.** Shown are the spectra at the end of back-exchange, i.e. time point 15 hr, in dissolution buffer containing 75% D_2_O. Spectra were recorded at 278K, to slow down back-exchange. In all spectra, the contour level is set to five times the noise level as estimated by Sparky. Variations in overall signal intensity are due to differences in protein concentration.
(TIF)

Table S1 **Amino acid selective ^15^N-labeled samples of ncTom40 and their contribution to the H/D exchange experiments.**
(DOC)
