# Supplementary figures and images for: NMR-Based Detection of Hydrogen/Deuterium Exchange in Liposome-Embedded Membrane Proteins
Source: PLoS One. 2014 Nov 6;9(11):e112374. doi: 10.1371/journal.pone.0112374 (PMC4223039; doi:10.1371/journal.pone.0112374)

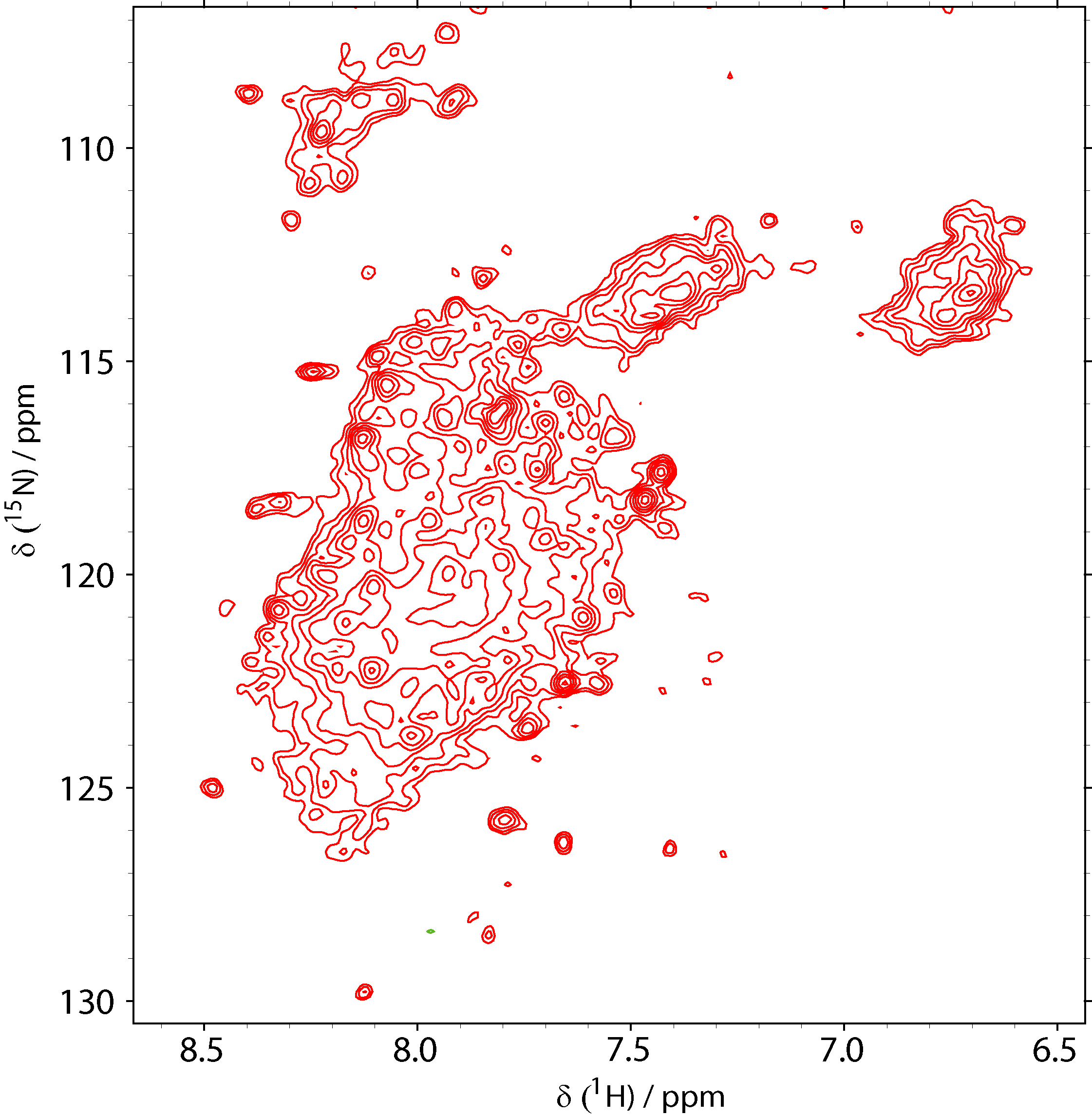

Supplement: Figure S1 — Two-dimensional [1H,15N]-HSQC spectrum of uniformly 15N-labeled ncTom40 reconstituted into lauryldimethylamineoxide. (TIF) [file pone.0112374.s001.tif]

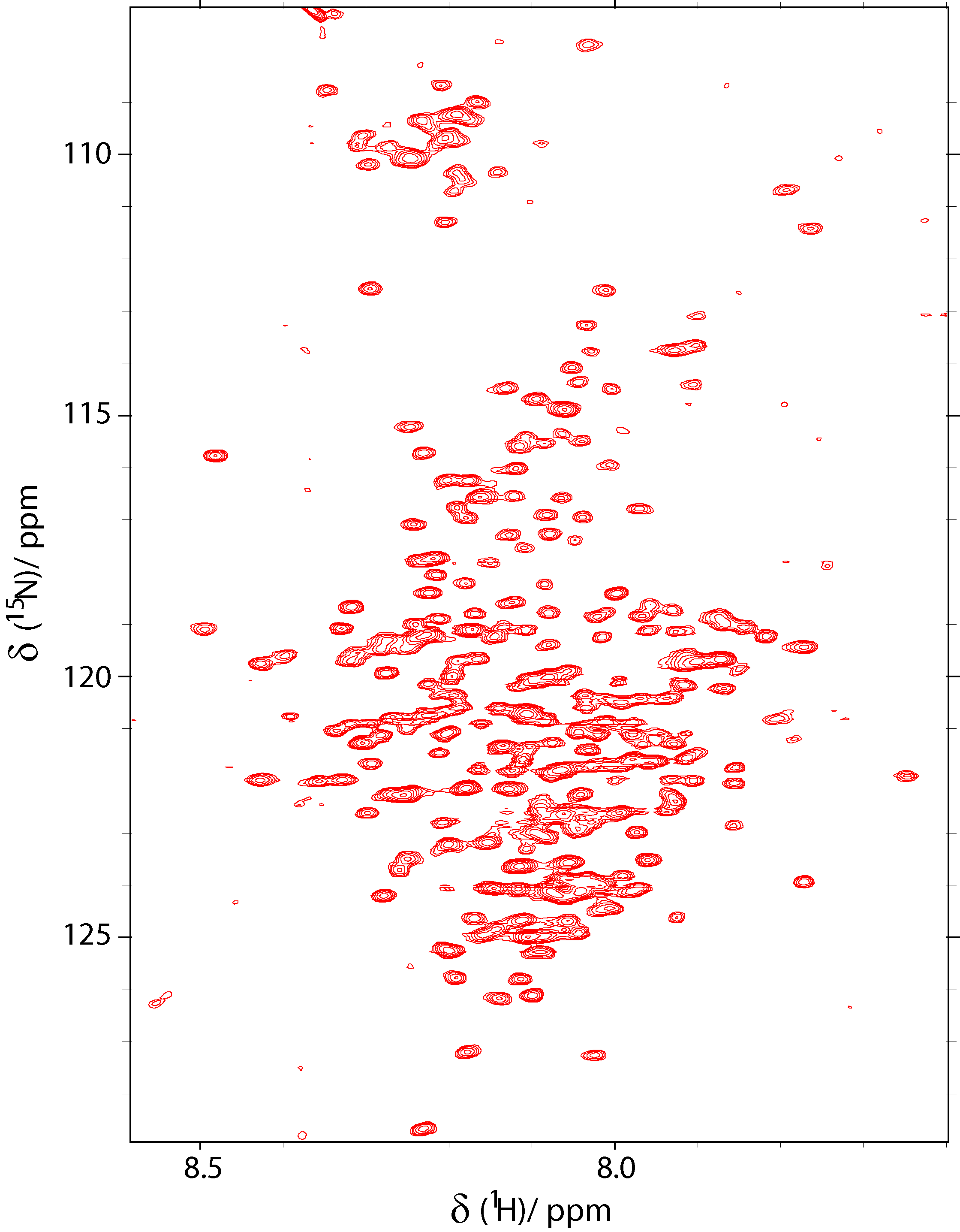

Supplement: Figure S2 — Two-dimensional [1H,15N]-HSQC spectrum of uniformly 15N-labeled ncTom40 in dissolution buffer containing 4M GdnSCN, 0.4% formic acid. (TIF) [file pone.0112374.s002.tif]

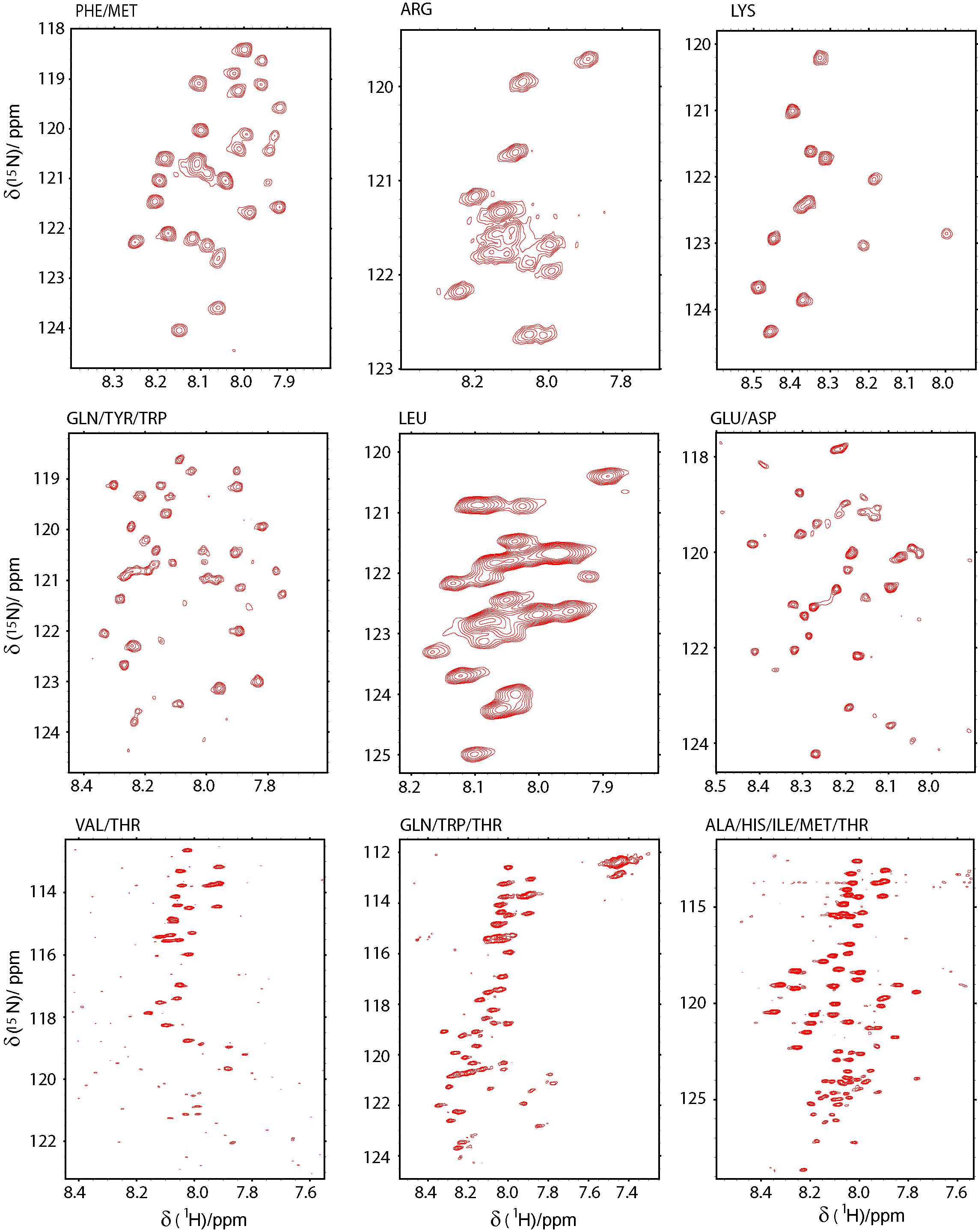

Supplement: Figure S3 — [1H,15N]-HSQC spectra of ncTom40 with amino acid selective 15N-labeling. Shown are the spectra at the end of back-exchange, i.e. time point 15 hr, in dissolution buffer containing 75% D2O. Spectra were recorded at 278K, to slow down back-exchange. In all spectra, the contour level is set to five times the noise level as estimated by Sparky. Variations in overall signal intensity are due to differences in protein concentration. (TIF) [file pone.0112374.s003.tif]
