# Supplementary material for: NMR-Based Detection of Hydrogen/Deuterium Exchange in Liposome-Embedded Membrane Proteins
Source: PLoS One. 2014 Nov 6;9(11):e112374. doi: 10.1371/journal.pone.0112374 (PMC4223039; doi:10.1371/journal.pone.0112374)
Supplement: Table S1 — Amino acid selective 15N-labeled samples of ncTom40 and their contribution to the H/D exchange experiments. (DOC) [file pone.0112374.s004.doc]

***Table S1:*** Amino acid selective ^15^N-labeled samples of ncTom40 and their contribution to the H/D exchange experiments.

| ^15^N-labeled amino acid | H/D exchange data |
| --- | --- |
| PHE/MET | 75% |
| LEU | 75% |
| ASN/ILE | 100% |
| Uniform | 75%, 100%(GLY/SER) |
| VAL/THR | 75%, 100% |
| GLN/TYR/TRP | 75% |
| LYS | 75%,100% |
| ARG | 75%,100% |
| ALA/HIS/ILE/MET/THR | 75%,100% |
| ASP/GLU | 75%, 100% |
| GLN/TRP/THR | 75%, 100% |
| PHE/GLU | 100% |
